# Mechanisms and regulation of iron trafficking across the capillary endothelial cells of the blood-brain barrier

**DOI:** 10.3389/fnmol.2015.00031

**Published:** 2015-07-14

**Authors:** Ryan C. McCarthy, Daniel J. Kosman

**Affiliations:** Department of Biochemistry, School of Medicine and Biomedical Sciences, University at BuffaloBuffalo, NY, USA

**Keywords:** blood-brain barrier, iron transport, cell communication, ferroxidase, Alzheimer’s disease

## Abstract

The transcellular trafficking of iron from the blood into the brain interstitium depends on iron uptake proteins in the apical membrane of brain microvascular capillary endothelial cells and efflux proteins at the basolateral, abluminal membrane. In this review, we discuss the three mechanisms by which these cells take-up iron from the blood and the sole mechanism by which they efflux this iron into the abluminal space. We then focus on the regulation of this efflux pathway by exocrine factors that are released from neighboring astrocytes. Also discussed are the cytokines secreted by capillary cells that regulate the expression of these glial cell signals. Among the interstitial factors that regulate iron efflux into the brain is the Amyloid precursor protein (APP). The role of this amyliodogenic species in brain iron metabolism is discussed. Last, we speculate on the potential relationship between iron transport at the blood-brain barrier and neurological disorders associated with iron mismanagement.

## Introduction

Organismal iron is a co-factor utilized for several specific enzymatic processes and, in higher eukaryotes, iron is essential for hemoglobin, myoglobin, neurotransmitter synthesis, myelination of neurons, and energy-producing redox reactions (Beard, 2003; Levi and Rovida, 2009; Todorich et al., 2009; Horowitz and Greenamyre, 2010; Chen and Paw, 2012). Therefore the brain, which is the most metabolically active organ in the body, has a high demand for iron and actively engages in maintaining appropriate amounts of the element within its confines. Brain iron maintenance is complex and involves both diurnal and regional regulation (Unger et al., 2009, 2014). The brain utilizes a detailed regulatory network involving cell-to-cell signaling and acute phase iron-regulatory proteins to transport iron from the blood, across the blood-brain barrier (BBB), and into the brain. However, before iron can enter the brain through the BBB it must first be acquired from the diet through a separate barrier system: the duodenal enterocyte barrier. Mechanisms of iron transport and regulation have been widely studied in the duodenal enterocytes and have been used as the foundation for studies examining the iron regulatory mechanisms at the BBB.

## Systemic Iron Uptake and Transport

At the duodenal enterocyte barrier, lumenal ferric iron (Fe^3+^) is reduced to ferrous iron (Fe^2+^) at the brush border (lumenal surface) of the duodenal enterocyte by an endogenous ferrireductase known as duodenal cytochrome b (Dcytb; McKie et al., 2001). Dcytb functions by transporting an electron from an endogenous cytosolic reductant, such as Nicotinamide adenine dinucleotide phosphate (NAD(P)H), to extracellular Fe^3+^. After reduction, Fe^2+^ crosses the lumenal surface of the duodenal enterocyte through divalent metal transporter 1 (DMT1; Fleming et al., 1999; Canonne-Hergaux et al., 2000; Oates et al., 2000). This function for DMT1 was indicated by phenotypic characterization of the Belgrade rat which contains a missense mutation in the *Nramp2* gene (encoding DMT1) and is impaired for intestinal iron absorption (Fleming et al., 1998). Once inside the enterocyte, iron can be utilized for assembly of Fe/S clusters and maturation of cytochromes and various non-heme iron enzymes;, stored in the iron storage protein ferritin; or exported out of the cell at the abluminal surface (blood-side).

Iron export from the abluminal surface of the duodenal enterocyte occurs via transport through the only known mammalian iron export protein, ferroportin (Fpn; Abboud and Haile, 2000; Donovan et al., 2000, 2005; McKie et al., 2000; Ganz, 2005; Han and Kim, 2007). The export of iron from Fpn (*IREG1/SLC40A1/MTP1*) requires the oxidation of that iron by an exocytoplasmic ferroxidase. Tethered to the extracellular surface of the duodenal enterocyte abluminal membrane is the multi-copper ferroxidase hephaestin (Hp; Vulpe et al., 1999; Anderson et al., 2002a; Han and Kim, 2007). Hp catalyzes the oxidation of Fe^2+^ to Fe^3+^ allowing for export of that iron from Fpn and its subsequent association with the iron-binding glycoprotein transferrin (Tf). A block in intestinal iron transport (specifically, a block in the efflux of iron from the enterocytes) was observed in the sex-linked anemia (sla) mouse (*Heph^sla^Heph^sla^*), which expresses a truncated form of Hp (Vulpe et al., 1999).

Now in the blood and bound to Tf, iron is transported safely throughout the bloodstream to be utilized by cells expressing the Tf receptor (TfR) on their surface. As high levels of iron accumulate in the serum, Tf becomes saturated and the increased levels of the diferric-Tf (holo-Tf) complex are sensed by liver hepatocytes. When iron replete, hepatocytes synthesize and secrete the peptide hormone hepcidin into serum (Anderson et al., 2002b; Lin et al., 2007; Anderson and Vulpe, 2009). The hepcidin peptide will enter the circulation and bind to its receptor Fpn; this interaction induces the internalization and subsequent lysosomal degradation of both Fpn and hepcidin (Nemeth et al., 2004; Ganz and Nemeth, 2012). Regulation of duodenal Fpn occurs post-translationally via this mechanism of hepcidin internalization. In addition, the major Fpn transcript contains an iron-responsive element (IRE) that is regulated by a set of iron-regulatory proteins (IRP1 and IRP2); these proteins bind to the IRE in response to low intracellular iron levels and prevent translational initiation of the transcript (Casey et al., 1988; Leipuviene and Theil, 2007). With fewer Fpn-expressing duodenal enterocytes, iron entry into the blood is diminished. However, duodenal enterocytes express also a 1b-isoform of Fpn which lacks an IRE in its 5′ untranslated region (UTR); thus, unlike many other iron handling gene products, the Fpn expression from this transcript bypasses the normal IRP-dependent repression of intestinal iron uptake (Zhang et al., 2009). This description defines the main regulatory axis for iron entry into the systemic circulation through the abluminal surface of the duodenal enterocyte barrier.

## Iron Mobilization from Blood to Brain: Mechanisms of Transport Across the BBB

Similar to the duodenal enterocyte barrier, iron entry into the brain is regulated at the BBB by a regulatory network involving multiple cell types and signaling molecules. The BBB is a barrier system coordinated by three different cell types: brain microvascular endothelial cells (BMVEC), astrocytes, and pericytes (Abbott et al., 2006). The pericytes of the BBB are thought to be involved in vaso-constriction and dilation of the blood vessel and will not be discussed further in this review. Here we will focus on the regulation of iron transport from the blood, through the BBB, and into the brain with the view that the BMVEC and astrocytes form a regulatory axis which modulates this process. We note the similarities between systemic iron regulation at the duodenal enterocyte barrier and what is known about the central nervous system’s (CNS) iron regulatory mechanisms as they pertain to the BBB.

**Figure 1:**
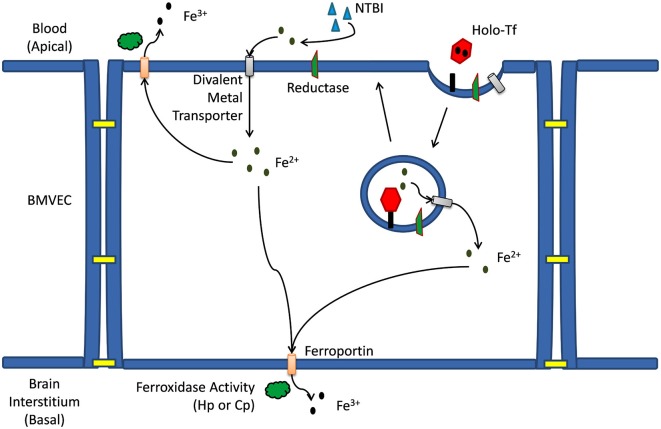
**Iron trafficking mechanisms across a brain microvascular endothelial cell.** Mechanism(s) of iron trafficking depicted include transferrin iron uptake (TBI uptake) and non-transferrin bound iron uptake (NTBI uptake). Whether accumulating in the cytosol as a result of release from the endosome, or as a result of transport across the apical membrane *via* a divalent metal ion transporter, the Fe^2+^ can efflux from the capillary cell back into the blood, or at the basal membrane into the brain interstitium. This efflux is *via* the ferrous iron transporter, ferroportin (Fpn); iron release from Fpn into the extracellular milieu depends on the ferrous iron oxidation activity (ferroxidase activity) by a multi-copper oxidase, either ceruloplasmin (Cp) or hephaestin (Hp).

The BMVEC of the BBB are a polarized cell type exposed to two distinct environments on their opposing apical and basal surfaces (Figure 1). The BMVEC are exposed to circulating holo-Tf (TBI) and non-Tf bound iron (NTBI) species at their apical (blood) surface. BMVEC express approximately 100,000 Tf receptors per cell on their plasma membrane and readily accumulate iron from holo-Tf via a canonical Tf-TfR mediated uptake pathway as evidenced by *in vitro* examination of hBMVEC Tf-iron uptake (Raub and Newton, 1991; McCarthy and Kosman, 2012, 2013).

Canonical Tf-iron uptake by BMVEC begins when holo-Tf binds to the TfR [affinity is ~10^22^ M^−^1 at pH 7.4 (Aisen et al., 1978)] on the apical surface of BMVEC resulting in a clathrin-dependent endocytosis of the Tf-TfR complex and the subsequent formation of an early endosome. Within the endosome iron will be reduced and released from Tf and will subsequently exit the endosome through a divalent cation transporter such as DMT1 (Figure 1; Fleming et al., 1998; Burdo et al., 2001; McCarthy and Kosman, 2012). For iron to be released from Tf in the endosome the electrochemical potential (*E*°) of the complex (<−500 mV Kraiter et al., 1998) needs to be made more positive than that of the endogenous reductants. The *E*° of the Tf-iron complex will be made more positive and iron will be effectively released from Tf in the endosome as a result of: (1) Tf binding to the TfR; (2) a reduction in endosomal pH; and (3) the presence of exogenous ligand (e.g., citrate; Byrne et al., 2010; Steere et al., 2010). The drop in endosomal pH [from 7.4 to 5.6 (Byrne et al., 2010)] occurs via the action of an endosomal H^+^-ATPase pumping protons into the endosome (Nelson and Harvey, 1999). The subsequent reduction of Fe^3+^ from Tf within the endosome occurs via the transfer of an electron from a cytosolic reductant such as NAD(P)H through an endosomal ferrireductase, and to the iron (Ohgami et al., 2005). Reductases identified in BMVEC include duodenal cytochrome b (Dcytb) and the six transmembrane epithelial antigen of the prostate 2 (Steap2; McCarthy and Kosman, 2012).

After the reduction of iron from Tf within the cycling endosome, the Fe^2+^ is then exported out of the endosome through a divalent cation transporter. DMT1 is the only divalent cation transporter to have been identified in BMVEC to date and has been localized with TfR in cycling endosomes within BMVEC (Burdo et al., 2001; Siddappa et al., 2002; McCarthy and Kosman, 2012; Du et al., 2014; Simpson et al., 2015); however, controversy concerning the expression and functional significance of DMT1 in BMVEC still remains (Moos et al., 2006; Skjørringe et al., 2012).

As an alternative to TBI uptake by BMVEC, NTBI uptake can occur at the apical surface of BMVEC (McCarthy and Kosman, 2012). NTBI in the blood may be bound to a variety of ligands (e.g., citrate, ATP, and albumin). When in spatial proximity to the apical surface of BMVEC, NTBI may be reduced to Fe^2+^ by a cell surface ferrireductase (Figure 1; McCarthy and Kosman, 2012). This ferrireduction has been suggested by the localization of non-heme ferrous iron at the apical surface of rat BMVEC (Meguro et al., 2008). Furthermore, we have demonstrated the cell-surface reduction of both TBI and NTBI *in vitro* using human BMVEC (hBMVEC) in combination with the colorimetric indicator ferrozine which reacts with the reduced Fe^2+^ to form a pink complex (McCarthy and Kosman, 2012). The ferrireductases which are functionally relevant to the reduction of iron at the BMVEC cell surface are unknown. As with the canonical Tf-TfR endosomal cycling pathway, cell-surface reduced Fe^2+^ can enter the cell through a divalent cation transporter (e.g., DMT1, Zip8, and Zip14). However, expression of Zip8 and Zip14 in BMVEC has not been examined.

Once ferrous iron enters the apical surface of the BMVEC through DMT1 it needs to be distributed throughout the cell and, relevant to this review, to the basal membrane of the cell to be exported into the brain. Upon import through DMT1, Fe^2+^ may be transferred to the DMT1-binding protein poly(rC)-binding protein 2 (PCBP2) which acts as a chaperone to deliver iron to iron-requiring enzymes, ferritin, and possibly even Fpn with which PCBP2 has been shown to interact (Shi et al., 2008; Leidgens et al., 2013; Frey et al., 2014; Lane and Richardson, 2014; Philpott and Ryu, 2014; Yanatori et al., 2014). The details of iron transport within the cytosol of the BMVEC have not been thoroughly investigated and the role of iron chaperones such as PCBP2 in this cell type remains unclear.

Irrespective of the chaperones involved, cytosolic iron will eventually be transferred to Fpn for export from the cell. Fpn is expressed by BMVEC and is localized to both their apical and basolateral surfaces (Wu et al., 2004; Enerson and Drewes, 2005; Yang et al., 2011; McCarthy and Kosman, 2013, 2014b; McCarthy et al., 2014; Simpson et al., 2015). This distribution of Fpn suggests iron may be returned to the circulation through Fpn localized on the apical surface of BMVEC; alternatively, iron can traffic into the brain through Fpn localized to the basolateral surface of these cells (Figure 1; McCarthy and Kosman, 2013, 2014b). Fpn is essential for brain development as Fpn knockout in mice is embryonic lethal while the Fpn trafficking mutant, *flatiron* (*ffe/ffe*), results in neural tube and forebrain development defects. Both phenotypes are due in part to the iron deficiency created by the lack of Fpn trafficking to the plasma membrane of cells (Donovan et al., 2005; Mao et al., 2010).

**Figure 2:**
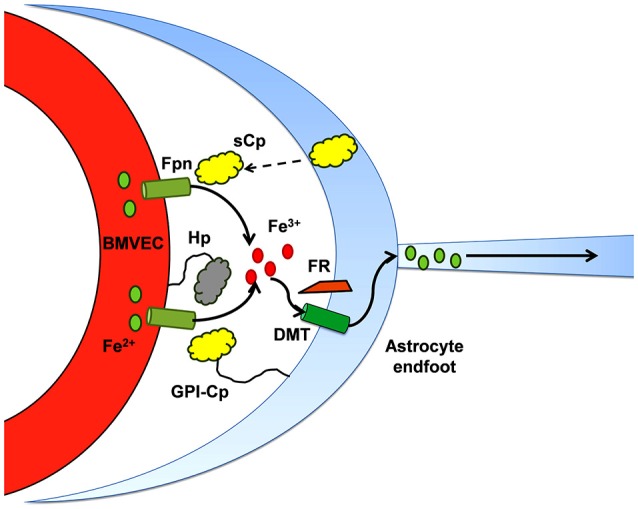
**Iron efflux from brain microcapillary endothelial cells depends on ferroxidase activity.** The ferroxidases, Hp and Cp, appear to support Fpn iron-efflux *via* two mechanisms which may not be separable: (1) in the presence of either *active* enzyme, the surface of expression of cellular Fpn increases, i.e., there are more Fpn transporters in the plasma membrane; and (2) the ferroxidase activity provided is proposed to “catalyze” the trafficking of Fe^2+^ through Fpn. This diagram illustrates that in the abluminal space of the BBB, in addition to Hp expressed by the BMVEC, sCp secreted by neighboring astrocytes plays a substantial role in supporting efflux of iron through the Fpn at the abluminal surface of BMVEC (McCarthy and Kosman, 2014b).

Similar to the duodenal enterocyte, iron efflux from BMVEC Fpn requires the action of an exocytoplasmic ferroxidase (Figure 2). In the case of hBMVEC, this ferroxidase activity may be provided by endogenous Hp or, when co-cultured with astrocytes, astrocyte-secreted soluble ceruloplasmin (sCp; McCarthy and Kosman, 2013, 2014b); expression of Hp by various cell types and tissues of the brain including BMVEC has been demonstrated (Qian et al., 2007; Wang et al., 2007; Cui et al., 2009; Yang et al., 2011). In culture, Hp is localized to the basolateral surface of hBMVEC where it likely acts in concert with basolateral Fpn to efflux iron from the cells (McCarthy and Kosman, 2013). Hp has been shown to enhance iron efflux from other cells of the CNS, i.e., oligodendrocytes. Oligodendrocytes cultured from *sla* mice (as noted, carrying a mutation in the hephaestin gene; Vulpe et al., 1999) are significantly deficient in their ability to efflux ^55^Fe in comparison to oligodendrocytes cultured from their wild type littermates (Schulz et al., 2011). The extent to which BMVEC Hp plays a role in glial cell iron handling in the adult rodent remains to be seen as the expression of Hp by these cells appears to be depleted in adulthood (Yang et al., 2011).

In *sla* mice, the Hp expressed exhibits reduced ferroxidase activity suggesting a compensatory mechanism enabling them to retain their ability to transport iron into the brain. Indeed, Cp has been shown to fulfil this role for Hp depletion in the white matter of the CNS from *sla* mice (Schulz et al., 2011). This compensatory ferroxidase activity can be provided by astrocyte-secreted sCp. The effect of astrocyte sCp on hBMVEC iron efflux has been thoroughly demonstrated *in vitro* using a three dimensional reconstruction of the BBB (McCarthy and Kosman, 2014b). Stimulation of iron efflux from hBMVEC by astrocyte-conditioned media was abrogated upon immunodepletion of sCp from that media (McCarthy and Kosman, 2014b). Therefore, it is likely that sCp from astrocytes provide adequate ferroxidase activity to stimulate the efflux of iron from Fpn at the basolateral surface of BMVEC. It should be noted however that Hp/Cp double-mutant mice still accumulate iron in the CNS suggesting further, yet undefined, compensatory mechanisms (Hahn et al., 2004; Hadziahmetovic et al., 2008; Schulz et al., 2011). Recently, Simpson et al demonstrated that iron efflux from rat BMVEC is enhanced by apo-Tf (Simpson et al., 2015). Whether or not apo-Tf compensates for BMVEC iron efflux in the Hp/Cp double-mutant mice is unclear. Perhaps a newly discovered ferroxidase, Zyklopen (Zp), fulfills this role (Danzeisen et al., 2002; Chen et al., 2010). Further examination of this hypothesis is required.

## Hepcidin Regulation of Iron Transport Across the BBB

Iron release from the abluminal surface of the duodenal enterocyte is regulated by the surface expression of Fpn. Fpn surface expression is differentially regulated by a balance of ferroxidase activity (e.g., Hp and/or sCp) and the peptide hormone hepcidin which is secreted by the liver in response to high levels of circulating holo-Tf. The basolateral surface of the BMVEC of the BBB is closed off from the circulation and therefore may not respond to circulating levels of sCp and/or hepcidin. Due to the lack of sCp/hepcidin permeability across the BBB and the high probability that the liver does not respond to brain iron levels, there must be a centralized system within the CNS responsible for regulating brain iron uptake through the BMVEC.

In keeping with the lessons learned from duodenal enterocyte Fpn regulation, several groups have examined the regulation of BMVEC Fpn by hepcidin. Hepcidin mRNA has been demonstrated to be widely expressed in mouse brain; furthermore, injection of hepcidin into the ventricles of the mouse brain induced a rapid depletion of overall brain Fpn suggesting hepcidin may indeed play a role in brain iron metabolism (Wang et al., 2010). Hadziahmetovic et al. demonstrated increased Fpn expression in the BMVEC of hepcidin null mouse mutants (Hadziahmetovic et al., 2011); this apparent effect of hepcidin on BMVEC Fpn has been corroborated (Du et al., 2014). Addition of hepcidin to cultured primary BMVEC reduces the expression of not only Fpn but also TfR and DMT1 suggesting that TfR and DMT1 transcripts are responding to elevated iron within the cell caused by Fpn depletion (i.e., decreased iron efflux; Du et al., 2014; Simpson et al., 2015).

While these data would indicate that hepcidin may be directly involved in BMVEC Fpn regulation, the cell type(s) from which the hepcidin peptide is generated within the brain has been examined. In 2014, we demonstrated that hepcidin, secreted from astrocytes, induced the internalization and ubiquitination of hBMVEC Fpn when seeded in close proximity in a model BBB system (McCarthy and Kosman, 2014b). This observation suggests that regulation of iron release from the basolateral surface of BMVEC is due to hepcidin secreted by neighboring glial cells, at the least. Whether the secretion of hepcidin from astrocytes is part of a feedback loop triggered by BMVEC-secreted iron has yet to be definitively determined. However, Simpson et al have recently demonstrated that basolateral BMVEC iron efflux can be increased by astrocyte-conditioned media from iron-depleted astrocytes. In contrast, media from iron-loaded astrocytes decreased basolateral BMVEC iron efflux (Simpson et al., 2015). These data are indicative of an existing feedback loop at the BBB that is modulated by astrocyte iron status.

Since both duodenal enterocytes and BMVEC constitute barrier systems, and the Fpn endogenous to each is regulated to a similar extent by hepcidin, it seems likely that they would both express the same isoform of Fpn, which is the Fpn1b isoform. The Rouault group performed immunohistochemistry and western blotting experiments assaying for Fpn in the brains of wild type (WT) and *IRP2*^−/−^ mice (Wu et al., 2004). They found no change in the relative expression of Fpn between the wild type and the *IRP2*^−/−^ mice maintained on either low or normal iron diets suggesting that the Fpn1b isoform, which does not respond to changes in cellular iron content (Zhang et al., 2009), may constitute the major isoform of Fpn expressed by the brain (Wu et al., 2004). If the Fpn1b isoform is the major player in iron efflux from cells of the brain, then the significance of brain hepcidin in regulating the cell surface expression of Fpn in the brain would increase dramatically. At this point however, it remains unclear whether BMVEC express the Fpn1a and/or the Fpn1b isoform.

## BMVEC–Astrocyte Signaling Network in Brain Iron Trafficking

**Figure 3:**
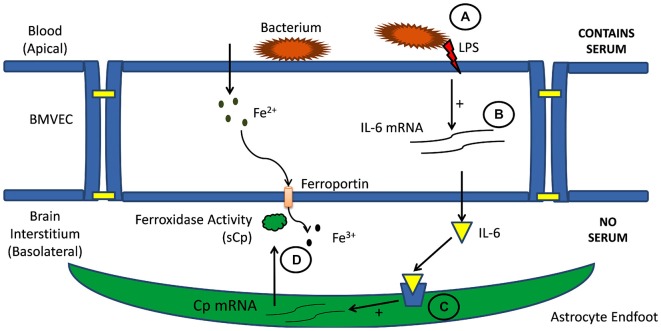
**Cell-cell signaling in brain iron uptake.** Illustrated is the signaling pathway from blood to BMVEC **(A)** and then through the abluminal space **(B)** to astrocytes neighboring BMVEC **(C)**. Circulating inflammatory signals stimulate interleukin expression and secretion from BMVEC. These cytokines stimulate astrocyte expression and secretion of sCp into the interstitium **(D)**. This sCp supports an increase of iron efflux from the adjacent BMVEC (McCarthy and Kosman, 2014a).

As noted above, glial cell hepcidin and sCp provide the regulatory signals responsible for managing iron efflux from BMVEC: sCp stimulates iron efflux by enhancing Fpn surface localization and catalyzing iron release; hepcidin knocks down Fpn surface localization and thereby reduces iron efflux. There is however an additional layer of complexity in regards to the regulation of iron transport at this cellular junction (Figure 3). That is, cytokine signaling from BMVEC to astrocytes seems to play a role in modulating the expression of astrocyte sCp (McCarthy and Kosman, 2014a). Specifically, astrocyte Cp gene expression increases in response to the signaling molecules interleukin-1β (IL-1β) and IL-6 (di Patti et al., 2004; Conley et al., 2005; Persichini et al., 2010; Sidhu et al., 2011; McCarthy and Kosman, 2014a). BMVEC have been shown to express and secret both IL-1β and IL-6 into their culture medium suggesting that BMVEC could directly influence the expression of astrocyte Cp (Podor et al., 1989; Sironi et al., 1989; Corsini et al., 1996; Frigerio et al., 1998; Wilson et al., 2007; McCarthy and Kosman, 2014a). Until recently however, the connection between BMVEC secretion of cytokines IL-6 and IL-1β and their induction of neighboring astrocyte Cp gene expression had not been drawn. Our lab has recently demonstrated that IL-1β and IL-6, secreted from hBMVEC, directly modulate the expression of astrocyte sCp which, in turn, acts on hBMVEC Fpn to enhance iron efflux from the cell (McCarthy and Kosman, 2014a). Based on these data, we suggest that hBMVEC have the capacity to modulate their own iron efflux through this intricate signaling network involving IL-1β and IL-6.

Interleukins such as IL-1β and IL-6 are often involved in the inflammatory response; therefore, their expression by hBMVEC and their subsequent downstream effect on astrocyte sCp gene expression are likely responsive to inflammatory signals (Figure 3). Indeed, addition of the bacterial endotoxin lipopolysaccharide (LPS) to the apical surface of hBMVEC increased the gene expression of both IL-1β and IL-6 and as well as the gene expression of sCp within astrocytes seeded on the basolateral side of the hBMVEC. However, data would indicate that only IL-1β plays a role in modulating neighboring astrocyte sCp gene expression in the presence of the inflammatory signaling molecule LPS (McCarthy and Kosman, 2014a). In addition to modulating astrocyte sCp levels, inflammatory cytokines (tumor necrosis factor alpha, IL-6, and LPS) have been shown to alter the expression of the iron regulatory proteins DMT1, Fpn, and hepcidin in cells of the CNS (Urrutia et al., 2013; Zhang et al., 2014). Regulation of these proteins by inflammatory stimuli were not investigated in endothelial cells.

An important question is whether the effect of LPS on astrocyte sCp production and increased brain iron import is a protective mechanism for the brain or if it is a mechanism by which the bacterium can disrupt the BBB and gain access to the brain. In regards to the first possibility, the brain may be protected by drawing iron out of the local environment of the blood, that is, withholding iron from the circulating pathogen. Alternatively, the microorganism may be creating a local environment of high oxidative stress incurred by excessive brain iron entry, thereby disrupting the barrier formed by the BMVEC. In this case the bacterium can readily enter the brain via paracellular trafficking around the distressed BMVEC. This would not be the first instance in which a pathogen alters the expression of iron regulatory proteins to support its proliferative capacity. The parasite *Leishmania amazonesis* infects macrophages and induces an up regulation in the macrophage hepcidin production (Ben-Othman et al., 2014). This, in turn, causes a decrease in macrophage Fpn on the cell surface providing more intracellular iron for the parasite to utilize for replication (Ben-Othman et al., 2014). Further investigation regarding the significance of BMVEC abluminal interleukin secretion as a result of receptor activation at the apical, blood membrane is clearly warranted.

## Amyloid Precursor Protein (APP) in Brain Iron Trafficking

There is abundant evidence for a correlation between brain iron trafficking and protein aggregation in Alzheimer’s disease (AD; Loef and Walach, 2012; Roberts et al., 2012; Greenough et al., 2013; Rouault, 2013; Schröder et al., 2013; Becerril-Ortega et al., 2014; Everett et al., 2014a,b). The underlying molecular basis of this apparent link between brain iron and the progression of this disease has not been delineated. The two salient features of AD pathophysiology are Aβ-peptide plaques and neuronal death. These indices of AD have been linked to iron by two hypotheses: (1) an intracellular iron-induced neuronal oxidative stress; or (2) an amyloidogenesis “catalyzed” by extracellular, interstitial iron. These two mechanistic proposals are not mutually exclusive. Indeed, some evidence suggests that redox active iron is produced in the course of Aβ aggregation (Everett et al., 2014a). Irrespective of the molecular details of disease progression, the data support a role for iron in this process.

One insight into this role follows from the question, what *is* the physiologic function of amyloid precursor protein (APP)? Certainly, this protein has not been selected for the purpose of causing dementia in the elderly. As a recent review noted: “Despite intensive research, the normal functions of the APP isoforms remain an enigma” (Nalivaeva and Turner, 2013). That one of its functions is related to iron homeostasis is strongly indicated by the fact that APP translation is regulated synchronously with ferritin *via* a 5′ UTR that contains an iron binding protein response element (Rogers et al., 2002, 2008). Thus, as cell iron increases the expression of APP increases as well. There is also evidence that indicates an increase in Beta secretase 1 (BACE1) expression and activity towards APP in PC12 cells treated with iron (Kim and Yoo, 2013). BACE1 is a protease involved in the extracellular cleavage of APP; subsequent cleavage of the remaining membrane-associated APP by γ-secretase forms (Aβ; Vassar et al., 1999). These data suggest a model in which increases in iron in the cells in the CNS would result in an increase in Aβ; reasonably, this alone could be the underlying connection between iron and amyloidogenesis.

**Figure 4:**
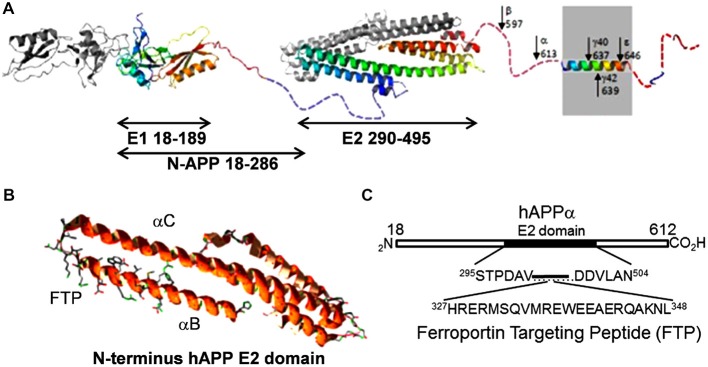
**The ferroportin binding element in the APP. (A)** A structural diagram depicting APP_695_, the predominant splice variant in the brain (modified from Muller and Wild, 2013). The E2 domain is indicated; note the first two C-terminal helices in this extracellular APP structural element. **(B)** Ribbon diagram of these first two helices (αBαC). The ferroportin binding motif is at the C-terminal end of αB; this motif is indicated by the included side chain display. Structure is based on PBD 3UMH. **(C)** Sequence of the ferroportin binding element designated Ferroportin Targeting Peptide (McCarthy et al., 2014).

**Figure 5:**
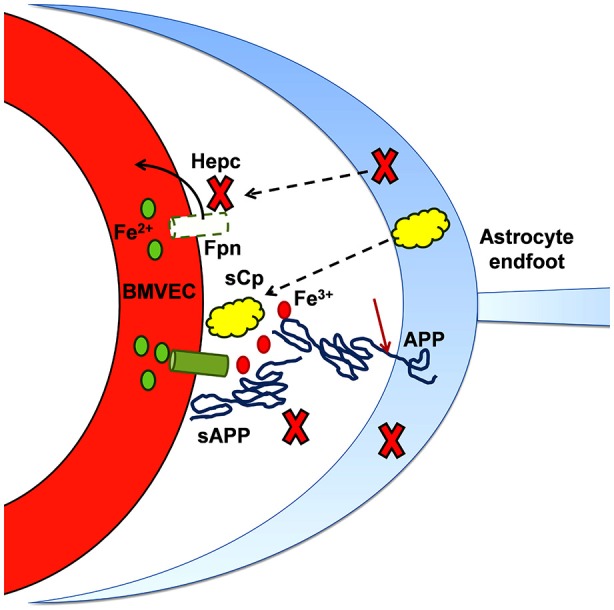
**Iron and the progression of amyloidogenesis.** As noted in the text, the expression of APP and (some of) the metalloproteases responsible for its cleavage into sAPP (and Aβ) is up-regulated by iron. This diagram highlights the role that sAPP plays in regulating the flux of iron into the abluminal space. In binding to BVMEC Fpn, sAPP potentiates the stabilization of Fpn at the basal surface that is otherwise provided by sCp. In addition, sAPP binding to Fpn *via* its ferroportin targeting peptide motif (FTP) likely antagonizes the inhibitory action of hepcidin (Hepc). In this model, sAPP, together with a ferroxidase (sCp), stimulates iron flux into the brain interstitium; in regards to brain pathology, this excess iron can support either or both oxidative stress and protein aggregation.

However, there is a second and likely more critical link, one suggested by the fact that sAPP binds to Fpn (Duce et al., 2010; McCarthy et al., 2014; Wong et al., 2014); APP may also exhibit a similar interaction (Wong et al., 2014). The binding site on Fpn is not known, however, the docking motif on APP has been identified. It is found within a short sequence at the C-terminal end of the αB helix in the protein’s E2 domain. The sequence of this peptide and its relation to the APP molecule is shown in Figure 4; illustrated is APP_695_, the most abundant splice form in the brain (Jacobsen et al., 1991). We have designated this peptide FTP for Ferroportin Targeting Peptide (McCarthy et al., 2014). In binding to Fpn, FTP stabilizes it in the membrane; this stabilization is likely due to a suppression of the hepcidin-independent retrograde recycling of Fpn into the cytoplasm that has been observed (Ganz, 2011). More significantly, however, is the apparent antagonism between FTP and hepcidin; this is illustrated in Figure 5. In this scenario, sAPP released from either glial cells or neurons in the neurovascular unit binds to Fpn in the basolateral membrane of hBMVEC and suppresses the normal regulation due to glial cell hepcidin as outlined above. This hypothesis for the connection between iron and Aβ-dependent neurodegeneration is under investigation.

## Synopsis of Iron Transport at the BBB and Neurological Disorders

Iron acts as a double-edged sword in living organisms. The ability to accept and donate electrons makes iron essential to life. Yet, when mismanaged, iron becomes highly toxic to cells; iron-induced generation of reactive oxygen species resulting in cell death is not uncommon (Rottkamp et al., 2001; Eaton and Qian, 2002; Altamura and Muckenthaler, 2009; Anderson and Wang, 2012). Neurological disorders have been attributed to both brain iron overload (i.e., Parkinson’s disease and AD) and brain iron deficiency (i.e., restless legs syndrome). In either case, the mismanagement of brain iron levels may involve the dysregulation of iron transport across the blood-brain barrier.

While it is known that iron accumulation is associated with both Alzheimer’s and Parkinson’s disease, the role that the BBB plays in modulating the increased brain iron load associated with these diseases is unclear (Gaasch et al., 2007; Altamura and Muckenthaler, 2009; Bolognin et al., 2011). In the case of AD, an accumulation of Aβ peptides adjacent to the endothelial cells of the BBB (cerebral amyloid angiogenesis) often precedes the neuron-associated Aβ peptide aggregates (Langer et al., 2011). A recent model we have proposed suggests that the sAPP fragment, generated during the cleavage events forming Aβ, may stabilize Fpn on the basolateral surface of BMVEC. This stabilization of BMVEC Fpn may allow for increased flux of iron into the brain. Subsequently, the iron may become associated with the local Aβ peptide aggregates leading to formation of neurotoxic oligomeric Aβ species and the progression of AD (Bolognin et al., 2011; McCarthy and Kosman, 2015).

Alternatively, it is possible that iron transport across the BBB is detrimentally effected by the chronic neuroinflammation that accompanies the progression of both Alzheimer’s and Parkinson’s disease. Chronic neuroinflammation and persistent microglia activation is a hallmark of these neurodegenerative disorders (Cahill et al., 2009; Maezawa et al., 2011; Cherry et al., 2014; Li et al., 2014; Morales et al., 2014). Excessive or mismanaged brain iron exacerbates the activation state of microglia resulting in excess production of reactive oxygen species and neurotoxicity (Urrutia et al., 2013; Thomsen et al., 2015). The elderly are more prone to systemic infections and stroke both of which may further exacerbate the inflammatory status of the brain ultimately leading to a breakdown in the BBB allowing for uncontrolled influx of iron into the brain parenchyma (Stankiewicz and Brass, 2009).

On the opposite end of the spectrum are patients with restless leg syndrome (RLS) which is associated with brain iron deficiency. Iron deficiency in these patients is hypothesized to cause dysfunction of dopaminergic systems resulting in the symptoms associated with the disease (Connor, 2008). Patients with RLS have increased transferrin and decreased ferritin levels in their cerebral spinal fluid (CSF) compared to control (Earley et al., 2000; Mizuno et al., 2005). This change in the iron management proteins ferritin and transferrin in the CSF could be the result of either increased brain iron efflux; alternatively this could be a compensatory mechanism to transport iron into the brain across the brain-CSF barrier *via* transferrin- or ferritin-mediated pathways. The role of the BBB in RLS remains to be thoroughly investigated.

## Conclusion

The studies reviewed in this article provide an overview of the pathways that support iron transport across the BMVEC of the BBB. While several iron handling proteins have been identified in BMVEC *in vivo*, their expression and their mechanism of action have been investigated mainly through the use of *in vitro* model systems that re-create the BMVEC and astrocyte interactions at the BBB. The mechanisms of iron regulation at the basolateral surface of BMVEC that are detailed in this review closely mimic those described for the duodenal barrier system. That is, both ferroxidase and hepcidin activity seem to create a natural balancing act regarding the control of iron efflux from the cell system. An additional factor in this balancing is coming into strong focus, namely APP and the protein/peptide products of its proteolysis. The normal physiology of the APP-hepcidin antagonism presumably maintains iron homeostasis in as much as this part of the balancing act has been selected for. With this perspective, the long-term pathophysiologic consequence of this mechanism may simply be the devastating consequence of the life-span doubling in the past 200 years.

Beyond the BBB, our lab has begun to understand the mechanism of iron uptake into primary hippocampal neurons. Our current understanding is that hippocampal neurons can accumulate both non-Tf-bound and Tf-bound substrates. Evidence suggests that Zip8 is the major ion channel for iron uptake into hippocampal neurons (Ji and Kosman, 2015). We hope to examine the influence of neuronal paracrine signaling on iron efflux from the basolateral surface of BMVEC. Neurons undoubtedly have the ability to influence the properties of the BBB and it is reasonable to propose that this influence could extend to regulating iron flux into the brain at the BBB. Whether the iron provided as substrate to neurons comes directly from BMVEC or only *via* astrocytes has yet to be determined; recent experiments have indicated that astrocytes act as a buffer for excess iron, thus preventing the over-accumulation of iron by neurons (Pelizzoni et al., 2013). Future experiments should investigate the role neurons play in drawing iron across the BBB into the brain.

## Conflict of Interest Statement

The authors declare that the research was conducted in the absence of any commercial or financial relationships that could be construed as a potential conflict of interest.
